# Prevalence of limited health literacy among patients with type 2 diabetes mellitus: A systematic review

**DOI:** 10.1371/journal.pone.0216402

**Published:** 2019-05-07

**Authors:** Adina Abdullah, Su May Liew, Hani Salim, Chirk Jenn Ng, Karuthan Chinna

**Affiliations:** 1 Department of Primary Care Medicine, University Malaya Primary Care Research Group (UMPCRG), Faculty of Medicine, University of Malaya, Kuala Lumpur, Malaysia; 2 Department of Family Medicine, Faculty of Medicine and Health Sciences, Universiti Putra Malaysia, Serdang, Selangor, Malaysia; 3 School of Medicine, Faculty of Health and Medical Sciences, Taylor’s University, Kuala Lumpur, Malaysia; University College London, UNITED KINGDOM

## Abstract

**Background:**

Health literacy (HL) skills are essential to enable self-management and shared decision-making in patients with type 2 diabetes mellitus (T2DM). Limited HL in these patients is associated with poorer outcomes. It is not clear what the burden of limited HL in patients with T2DM across countries and what factors influence it.

**Methods:**

A systematic review was conducted according to the PRISMA guidelines. The study protocol was registered with PROSPERO (CRD42017056150). We searched MEDLINE, EMBASE, PsycINFO, CINAHL and ERIC for articles published up to January 2017. Articles that measured HL levels in adult patients with T2DM; that used validated HL tools; and that were reported in English were included. Two reviewers assessed studies for eligibility and quality, and extracted the data. Prevalence of limited HL is calculated from the number of patients with less than adequate HL over the total number of patients with T2DM in the study. Meta-analysis and meta-regression analysis were conducted using the Open Meta-analyst software.

**Results:**

Twenty-nine studies involving 13,457 patients with T2DM from seven countries were included. In total, seven different HL measurement tools were used. The prevalence of limited HL ranged from 7.3% to 82%, lowest in Switzerland and the highest in Taiwan. Meta-regression analysis of all included studies showed the country of study (p<0.001), HL tool used (p = 0.002), and the country’s region (p<0.001) contributed to the variation findings. Thirteen studies in the USA measured functional HL. The pooled prevalence of inadequate functional HL among patients with T2DM in the USA was 28.9% (95% CI: 20.4–37.3), with high heterogeneity (I^2^ = 97.9%, p <0.001). Studies were done in the community as opposed to a hospital or primary care (p = 0.005) and populations with education level lower than high school education (p = 0.009) reported a higher prevalence of limited HL.

**Conclusion:**

The prevalence of limited HL in patients with T2DM varied widely between countries, HL tools used and the country’s region. Pooled prevalence showed nearly one in three patients with T2DM in the USA had limited functional HL. Interactions with healthcare providers and educational attainment were associated with reported of prevalence in the USA.

## Introduction

Globally, diabetes is a significant health problem. In 2017, the International Diabetes Federation estimated that 425 million people worldwide have diabetes and this number is expected to rise to 629 million in 2045. [1]Most people (90%) diagnosed with diabetes have type 2 diabetes. [2–4]The cause of type 2 diabetes is multifactorial but is related to unhealthy lifestyle activities like physical inactivity and poor diet. People with type 2 diabetes usually present late and about 30 to 80% of people with type 2 diabetes are still undiagnosed.[1] Late diagnosis of diabetes leads to diabetes complications like diabetes nephropathy and neuropathy at diagnosis.

Patients who are diagnosed are required to make daily decisions on healthcare and perform complex self-management activities to achieve disease control. Despite treatment advances and availability of clinical practice guidelines, only 30% of patients achieved glycaemic, blood pressure and cholesterol targets. The fact is that about 95% of diabetes care is provided by people with type 2 diabetes themselves.[5] Individual health literacy is fundamental to a person’s ability to manage their health and make appropriate health decisions.Health literacy is linked to literacy and entails people’s knowledge, motivation and competences to access, understand, appraise, and apply health information in order to make judgments and take decisions in everyday life concerning healthcare, disease prevention and health promotion to maintain or improve quality of life during the life course.[6] People with limited HL had been shown to have less health-related knowledge and reported poorer health status. [7]

Patients with type 2 diabetes and limited health literacy often cannot read medication labels accurately, may take medication incorrectly, may not understand consent forms, and generally have difficulty understanding print instructions for follow-up care and reading health advisories or warnings. [8] A recent review on health literacy and health outcomes in patients with T2DM concluded that there is consistent evidence to suggest a positive association between health literacy and diabetes knowledge. [9] Likewise, there is likely sufficient evidence to support a positive relationship between health literacy and self-care activities. [10] On the other hand, the evidence for an association between health literacy and clinical indicators was mixed. [11] The effect of health literacy on glycaemic control may be mediated by co-founders such as social support and self-efficacy. [12,13]

Patients with T2DM and limited HL also have less knowledge,[14] less medication adherence [15]and spend more on medications [16]. These patients also have poorer patient-doctor communications and participate less in decision-making. [17] Furthermore, interventions such as an educational intervention addressing HL and intensive diabetes self-management training adapted for patients with limited HL have been shown to improve diabetes outcomes. [18,19]

Recognising the problems associated with limited HL, some countries have proceeded to measure the burden of limited HL in their general populations and developed policies based on their findings. [20] This is exemplified by the publication of policy documents like the European Commission White Paper entitled ‘Together for health’, [21] the United States of America’s (USA) Department of Health’s ‘National Action Plan to Improve Health Literacy’ [22]and the WHO publication of ‘Health Literacy: the solid facts’. [23]

Several primary studies that looked at HL levels in patients with T2DM noted that a low proportion of patients with T2DM had adequate HL level, with reported prevalence ranging from 15 to 40%. Many of these studies were done in developed western countries like the USA and the UK. [24–26] However, there has been limited explanation of the observed differences in the prevalence and there was no effort to look at this problem globally.

There is much to understand by reviewing and summarising the burden of limited HL in patients with T2DM at the global level. Global prevalence data would enable governments, policy makers and healthcare practitioners to estimate the size of the problem, compare performances between countries and learn from countries with best practices and policies. In this review, we aim to summarise and report on current published evidence on the prevalence of limited HL in patients with T2DM globally and on the factors that are associated with the heterogeneity in the reported prevalence

## Materials and methods

The review protocol was registered with the international prospective register of systematic reviews, PROSPERO. (CRD42017056150)

### Data sources

We systematically searched five electronic databases (Medline, EMBASE, PsychInfo, CINHAL and ERIC) from the database inception up to January 2017. The definition of HL used in this review is by Sorensen et al. [6] This definition includes concepts such as numeracy, health education, health promotion, patient understanding and comprehension. The search strategy used keywords that encompassed these concepts and the search terms used for Medline are presented in S1 Table. The search terms were adapted for use in all the five databases. The search was limited to articles in the English language.

### Study selection: Inclusion and exclusion criteria

Studies that reported levels of HL in a population of patients with T2DM were included. There was no limitation on the study designs but we excluded data presented in conference proceedings, editorials and abstracts. Two reviewers (AA and HS) who are experienced primary care researchers and physicians, screened titles and abstracts for relevance and also performed the data extraction. Disagreements were resolved by consensus. Full texts of potentially relevant studies were searched and assessed for eligibility.

### Quality assessment and data extraction

We performed a quality assessment on the included studies using a critical appraisal checklist developed by the Joanna Briggs Institute (JBI) for systematic reviews of prevalence data. [27] The purpose of the quality analysis was to determine the extent to which the included study had addressed the possibility of bias in its design, conduct and analysis. The same reviewers independently carried out the quality assessment using the nine questions posed by the checklist.

Data from the included studies were extracted using a predetermined data extraction form. Data extracted on study information were the year of study, study design, the name of HL tool used, study settings (e.g. country, study site), population details (e.g., age, ethnicity, gender, education level, socioeconomic status) and prevalence of limited HL. Limited HL is defined as any level of literacy below adequate HL; some prevalence values were extracted from the data reported in the manuscript but some were calculated by the reviewers by dividing the number of patients with type 2 diabetes and limited HL over the total number of patients with type 2 diabetes in the study

### Data synthesis

The studies were grouped by country to allow for inter- and intra-country comparisons. The I^2^ statistics were calculated to measure the degree of heterogeneity between studies and a value above 75% indicates high heterogeneity. We aimed to include all studies in a meta-analysis using a random effect model to account for heterogeneity. We utilised the OpenMetaAnalyst software [28] (downloaded from http://www.cebm.brown.edu/openmeta/).

Potential factors influencing the prevalence estimates were determined using a meta-regression analysis. Factors included as co-variates were studies’ characteristics: study settings, the mean age of participants, the proportion of female participants, the proportion of African-American participants and the proportion of participants with more than high school. These factors were identified as a *priori* from previous literature and extracted from included studies during the data extraction stage.

## Results

**Fig 1:**
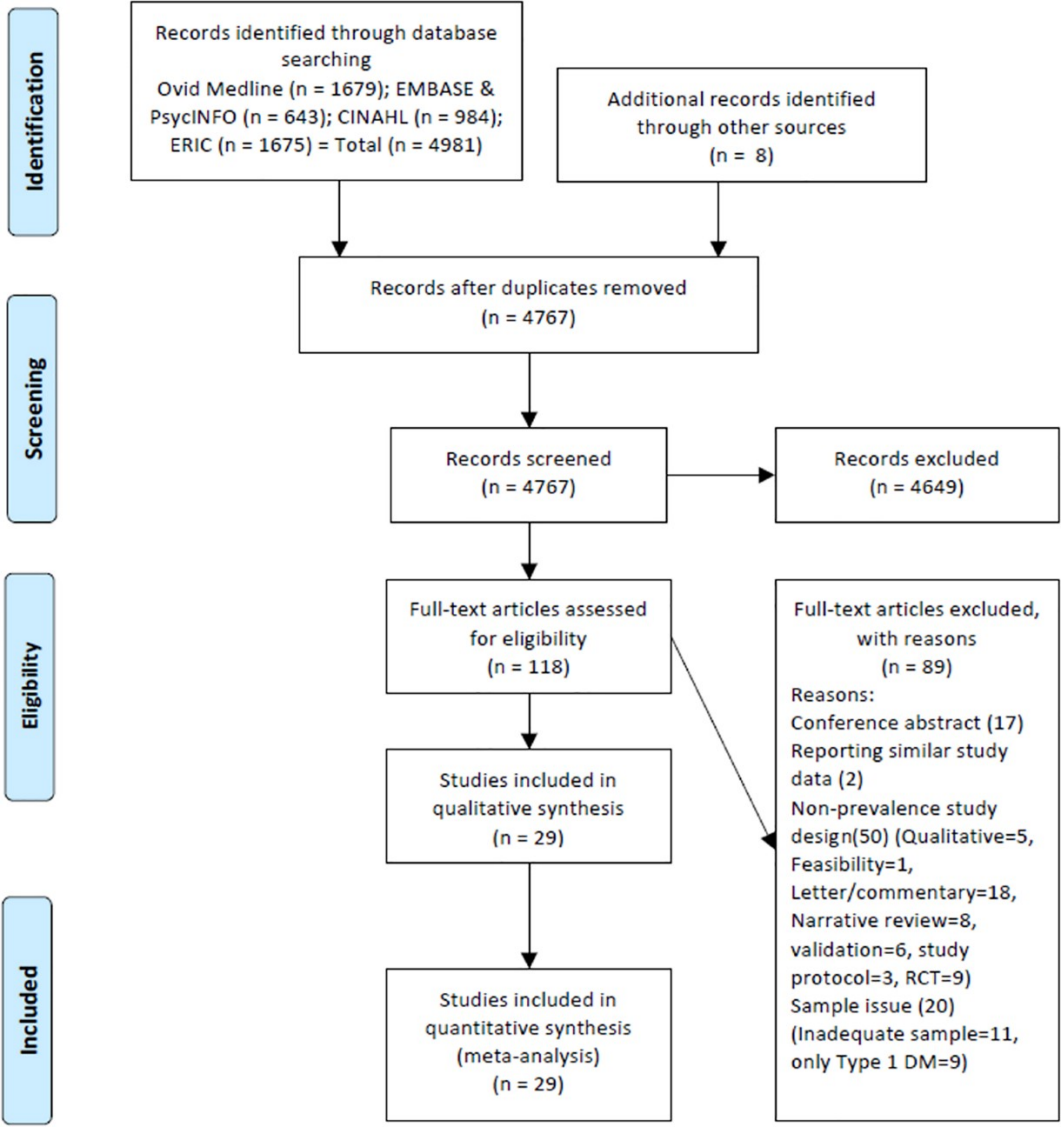
PRISMA diagram of the articles selection process. (Refer Fig 1_PRISMA diagram of article selection process.TIFF).

The search of databases yielded 4,981 potentially relevant studies and eight were found from citation tracking. After the removal of duplicates, 4,767 articles remained. Titles and abstracts were screened for relevance, and from these, 118 studies were included for full text review. Of these 118 studies, 89 were excluded. The final number of included studies was 29. PRISMA flow diagram is presented in Fig 1.

### Included studies

We extracted prevalence data from all 29 studies. Details of the included studies are presented in Table 1. Most (n = 24) of the included studies used a cross-sectional study design; three were longitudinal studies,[29–31] one was a cohort [32] and one was an interventional study [33]. The prevalence data of this review included 13,457 patients with T2DM. Of the 29 studies, 18 studies were conducted in the USA [25,26,31–46] and the rest were done in Canada (2 studies), [29,30] Brazil (2 studies), [47,48] ] Switzerland (2 studies), [49,50] Netherlands (1 study), [51]Marshall Island (1 study), [52] South Korea (1 study) [53] and Taiwan (2 studies) [54,55] ]. The studies included in this review were published as early as 2000 and the latest in 2014.

**Table 1:** Characteristics of included studies. (Refer Table 1 Characteristics of included studies).

Country	Authors (year)	Year	Sample size	Main aims	Study design	Setting	Tool	Participants	Prevalence: % (n/N)
Brazil	De Castro, S. H., et al (2014)	NR	150	To assess the frequency of full and functional health illiteracy	Cross-sectional	Hospital outpatient	s-TOFHLA	Mean age = 58.5 years (SD 9.8), 52.4%, female, 28.4%—less than high school education.	26.7% (40/150)
Brazil	Souza, J. G., et al (2014)	2012	225	To investigate the relationship between functional health literacy and glycaemic control in a sample of older patients	Cross-sectional	Hospital outpatient	SAHLPA-18	Mean age = 75.9 years (SD 6.2), 69.8%, female, 82.9%—having less than a high-school diploma.	45% (58/129)
Canada	Al Sayah, F., et al (2015)	NR	154	To examine the relationship of inadequate health literacy (HL) with changes in depressive symptoms, health-related quality of life and cardiometabolic outcomes in patients recently screened positive for depression.	Longitudinal	Primary care clinics	BHLS	Mean age = 58.1 years (SD 9.4), 55.8%, female, 13.7%—less than high school	15.6% (24/154)
Canada	Sayah, F. A., et al (2016)	2013	1948	To examine the association of health literacy (HL) with changes in health-related quality of life (HRQL)	Longitudinal	Primary care clinics	BHLS	Mean age = 65.6 years (SD 11.4), 45%, female, 14.2%—less than high school education.	12.6% (244/ 1948)
Marshall Island	Bohanny, W. M., et al (2013)	2009	150	To explore the relationships among health literacy, self-efficacy, and self-care behaviors	Cross-sectional study	Primary care clinics	s-TOFHLA	Mean age = 52.7 years (SD 10.5), 53.3%, female, 44%—less than high school	24% (36/150)
South Korea	Kim, S. H. (2009)	2007	103	To investigate the relationships of health literacy to chronic medical conditions and the functional health status	Cross-sectional study	Community based	Korean Functional Health Literacy test	Mean age = 67.2 years, 63.3%, female with limited literacy.	71.7% (43/60)
Switzerland	Franzen, J., et al (2014)	2011	493	To measure functional HL among persons having type 2 diabetes and to investigate the relationship between functional HL and health care costs and utilization.	Cross-sectional study	Insurer's database	BHLS	Mean age = 67.5 years, 51.5% belongs to 65–70 year-old group, n = 391, 32.7%, female	7.3% (36/493)
Switzerland	Mantwill, S., et al (2015)	2012	391	To determine the relationship between health literacy and three years of medication costs	Cross-sectional study	Insurer's database	BHLS	Mean age = 63.8 years (SD 6.1), 32.2%, female, 13.1%—less than high school education.	8.7% (34/391)
Taiwan	Chen, G. D., et al (2014)	2012	467	To demonstrate the interaction of health literacy and understanding of health education and instructions in achieving glycemic control	Cross-sectional study	Hospital outpatient	MHLS	Mean age = 68.3 years (SD 7.4), 70.2%, female with limited literacy, 61.5%—less than compulsory education	82% (383/467)
Taiwan	Tseng, H.-M., et al (2017)	NR	232	To explore the mechanisms through which HL is associated with the health outcome of diabetic care.	Cross-sectional study	Hospital outpatient	NVS	Mean age = 58.1 years (SD 9.49), 44.8%, female, 90.1%—secondary education and less	76.3% (177/232)
Netherlands	van der Heide, I., et al (2014)	2010	1941	the aim of the present study is to investigate whether diabetes knowledge can account for part of the relation between health literacy and diabetes self-management behavior	Cross-sectional study	Primary care clinics	BHLS	65–74 years group (31.7%), 49.6%, female, 44.9% low level of education	9.7% (167/1714)
United States of America (USA)	Schillinger, D., et al (2002)	2000	408	To examine the association between health literacy and diabetes outcomes	Cross-sectional study	Primary care clinics	s-TOFHLA	Mean age = 62.7 years (SD 10.9), 58%, female, 46%—some high school education or less	51.5% (210/408)
United States of America (USA)	Rothman, R., et al (2004)	2000	111	To examine the role of literacy in patients with poorly controlled diabetes who were participating in a diabetes management program that included low-literacy-oriented intervention .	Cross-sectional study	Hospital internal medicine clinic	REALM	Mean age = 60 years, 56%, female has limited health literacy, 82%—less than high school education	55% (61/111)
United States of America (USA)	Laramee AS, et al (2007)	2005	998	To determine the prevalence of limited literacy in diabetic patients with heart failure (HF) compared to those with diabetes and no HF.	Cross-sectional study	Primary care clinics	s-TOFHLA	Mean age = 65 years (22–93), 54%, female, 25%—less than high school graduate.	17.1% (171/998)
United States of America (USA)	DeWalt, D. A., et al (2007)	2005	268	To examine the relationship between literacy and trust, self-efficacy, and participation in medical decision making	Cross-sectional study	Hospital outpatient	REALM	Mean age = 62 years (SD 10), 57%, female with limited health literacy .	19.8% (53/268)
United States of America (USA)	Aikens JE, Piette JD. (2009)	2007	803	To determine how patients’ beliefs about antihyperglycemic and antihypertensive medications relate to medication underuse and health status.	Cross-sectional study	Primary care clinics	BHLS	Mean age = 55.3 years (SD 11.8), 61.6%, female, 21.6%—less than high school	37.2% (299/803)
United States of America (USA)	Jeppesen KM, et al (2009)	2007	225	To identify questions that could best indicate to a clinician that a patient may have low or marginal health literacy	Cross-sectional study	Primary care clinics	s-TOFHLA	Mean age = 53.8 years (SD 12.8), 68.4%, female, 44.9%—less than high school education.	15.1% (34/225)
United States of America (USA)	Mancuso, J. M. (2010)	NR	102	To examine if health literacy and patient trust in one’s health-care provider impact glycemic control in an uninsured population	Cross-sectional study	Primary care clinics	TOFHLA	Mean age = 52 years (SD 9.1), 60.8%, female, 33.3%—Less than high school education.	36.3% (37/102)
United States of America (USA)	Mbaezue N, et al (2010)	2005	189	To examine the relationship between health literacy and self-monitoring of blood glucose (SMBG)	Cross-sectional study	Hospital-based clinic	s-TOFHLA	Mean age = 51.2 years (SD 10.0), 58.7%, female, 32.3%—less than high school education.	39.1% (74/189)
United States of America (USA)	Wallace, A. S., et al (2010)	2008	195	To examine whether demographic characteristics, insurance status, literacy, duration of diabetes, and intensity of care management were associated with PACIC ratings	Cross-sectional study	Hospital diabetes clinic	s-TOFHLA	Mean age = 58 years (range: 23–85), 64%, female, 34%—Less than high school education.	29.3% (61/208)
United States of America (USA)	Bauer, A. M., et al (2013)	2006	1366	To determine whether health literacy limitations are associated with poorer antidepressant medication adherence.	Cohort study	Insurer’s database	BHLS	Mean age = 58.7 years (SD 10.5), 59.9%, female with limited Health literacy, 28.1%—less than high school	72% (984/1366)
United States of America (USA)	Bowen, M. E., et al (2013)	2009	144	To describe the association among numeracy, total energy,and macronutrient intake	Cross-sectional study	Primary care clinics	REALM	Median age = 56 years, 53%, female, 26%—high school education or less	11.1% (16/144)
United States of America (USA)	Morris, N. S., et al (2013)	2007	751	To evaluate the stability of health literacy over time	Longitudinal study	Primary care clinics	s-TOFHLA	12% belong to 70 years old age group, 53%, female with limited health literacy, 70%—Some high school education.	12.8% (96/751)
United States of America (USA)	Mayberry, L. S., et al (2014)	2012	183	To assess whether obstructive family behaviors had a stronger relationship with worse glycemic control among patients with limited HL than among those with adequate Health literacy	Cross-sectional study	Hospital outpatient	s-TOFHLA	Mean age = 51.2 years (SD 10.6), 70%, female, 64%—less than high school education	26.2% (48/183)
United States of America (USA)	Thurston, M. M., et al (2015)	2013	288	To determine (1) if a relationship exists between health literacy and self-reported or objectively measured medication adherence and (2) which aspect or aspects of medication nonadherence are most associated with health literacy.	Cross-sectional study	Primary care clinics	s-TOFHLA	Mean age = 54.4 years (SD 10.3), 56.8%, female, 64.6%—less than high school education	32.8% (63/192)
United States of America (USA)	Sayah, F. A., et al (2015)	2010	343	To examine the associations between inadequate health literacy and behavioral and cardiometabolic parameters	Cross-sectional study	Primary care clinics	BHLS	Mean age = 57.4 years (SD 10.11), 68%, female, 25%—less than high school education	24% (82/343)
United States of America (USA)	Goonesekera, S. D., et al (2015)	2012	682	To examine racial/ethnic differences in receipt of hypoglycaemic medications and glycaemic control	Cross-sectional study	Community based	s-TOFHLA	56% belongs to less than 65 years old group, 51%, female, 18%—less than high school.	51.5% (351/682)
United States of America (USA)	Fan, J. H., et al (2016)	2014	208	To investigate the relationship between health literacy and overall medication nonadherence, unintentional nonadherence, and intentional nonadherence.	Cross-sectional study	Primary care clinics	BHLS	Mean age = 53 years (SD10.9), 70.9%, female, 19%—had less than a high school education	63.5% (132/208)
United States of America (USA)	Nelson, L. A., et al (2016)	NR	80	To examine the relationship between patient factors and engagement in an mHealth medication adherence promotion intervention for low-income adults	Intervention study	Hospital outpatient	BHLS	Mean age = 50.1 years (SD 10.5), 54%, female, 56.3%—less than a high school degree	46.3% (37/80)

The study with the highest prevalence of limited HL (82%) was conducted in 2012 with the aim to demonstrate the interaction of health literacy and understanding of health education and instructions in achieving glycaemic control among 467 Taiwanese patients with T2DM. This cross-sectional study used the Mandarin Health Literacy Scale (MHLS). The mean age of the participants was 68.3 years (SD 7.4), 70.2% of participants with limited HL were female and 61.5% had less than compulsory education. [54] The lowest prevalence of limited HL (7.3%) was reported in 2011 with the aim to measure functional HL among persons having type 2 diabetes and to investigate the relationship between functional HL and health care costs and utilization in Switzerland. This cross-sectional study used Chew’s Brief Health Literacy Screener. The mean age of participants in this study was 67.5 years with 51.5% belonged to the 65 to 70-year-old group and 32.7% was female.[50]

**Fig 2:**
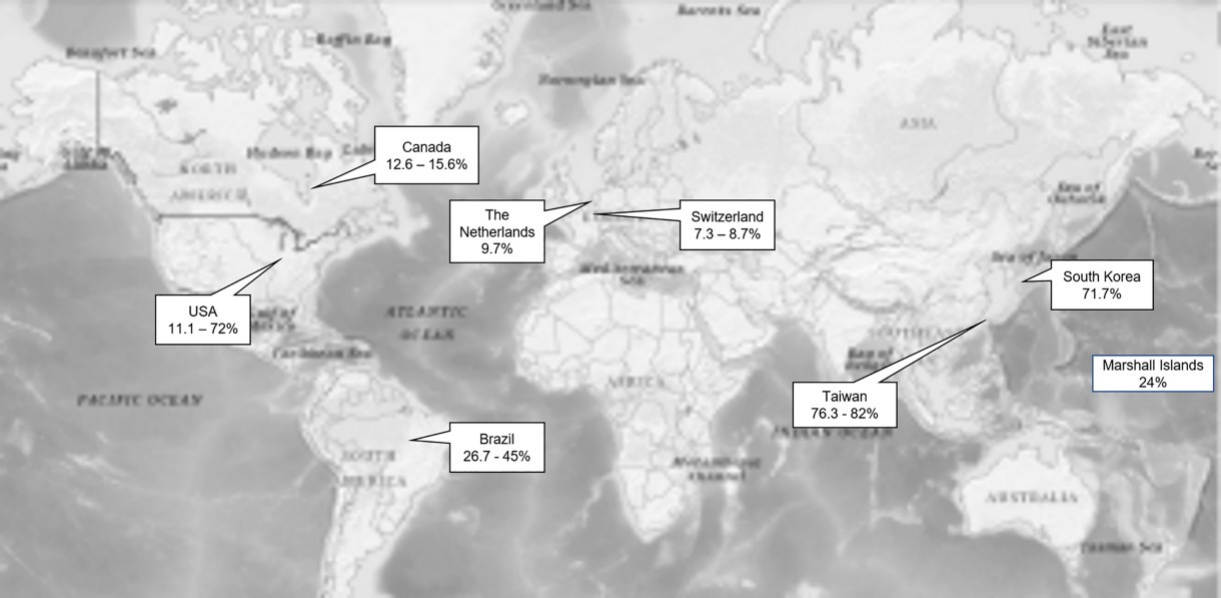
Worldwide prevalence of limited HL in patients with type 2 DM. (Refer Fig 2_Worldwide prevalence of limited HL.TIFF).

The country’s prevalence of limited HL (or the range if more than one study reported in the country) is displayed on the global map in Fig 2.

### Quality assessment

**Fig 3:**
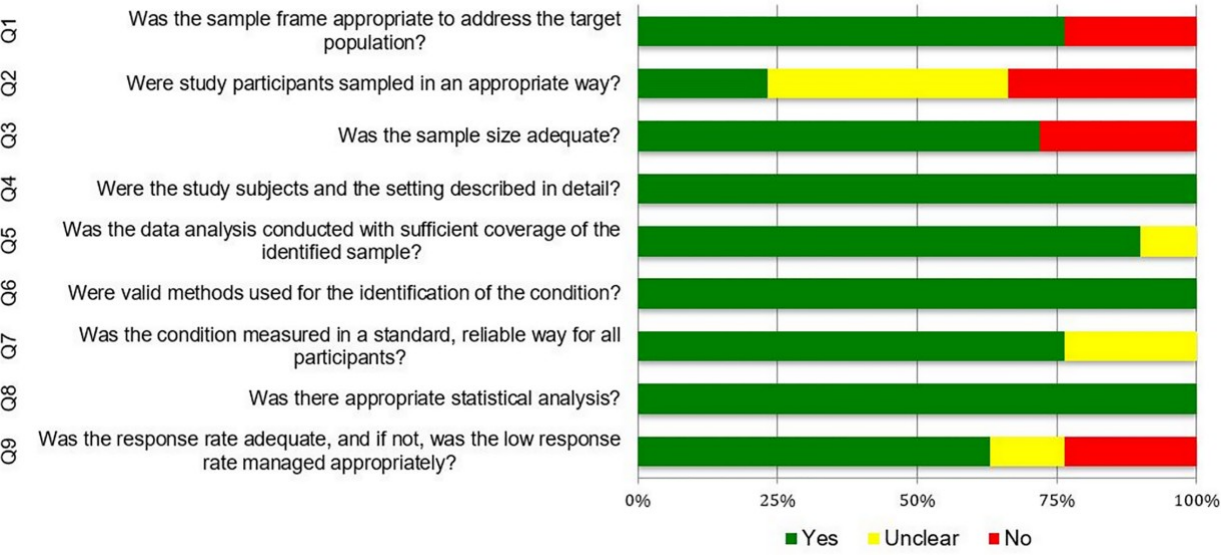
Quality assessment of included studies. (Refer Fig 3 Quality assessment of included studies.TIFF).

Based on the JBI critical appraisal checklist for prevalence studies, most studies had limitations in study quality (Fig 3). Only 10% (3/29) fulfilled all the assessment criteria. Most, 41% (12/29) did not meet the sampling approach criterion. Many of these studies used convenience sampling, which limits the generalisability of the reported prevalence. Other criteria with 72% (21/29) fulfilment are the sample size, use of appropriate measurement tool and detailed reporting of the participants and study setting.

### HL measurement tools

Methods of HL measurement differed between studies and measured either one aspect of HL like functional literacy, or several domains of health literacy. Earlier studies used functional health literacy tools mainly. These tools were usually objective tools and measured only the reading and comprehension abilities. Tools used to measure functional HL in the included studies were the Test of Functional Health Literacy in Adults (TOFHLA and its abbreviated version, s-TOFLA) (12 studies); Rapid Estimate of Adult Literacy in Medicine (REALM) (3 studies) and derivatives of REALM such as 18-item Short Assessment of Health Literacy for Portuguese-speaking Adults (SAHLPA-18) (1 study); Newest Vital Sign (NVS) (1 study) and the Korean Functional Health Literacy test (KFHL) (1 study). Other tools used are self-reported which measured a multidimensional concept of health literacy such as Chew’s Brief Health Literacy Screener (BHLS) (10 studies) and the Mandarin Health Literacy Scale (MHLS) (1 study). The definition of limited HL varied between the included studies depending on the HL measurement tools. Some tools used five categories whereas others used four categories to group patients’ HL levels.

### The pooled prevalence of limited HL: A meta-analysis

The pooled global prevalence of limited HL was 34.3% (95% CI: 25.8–42.8). Meta-analysis of all the studies yielded high heterogeneity (I^2^ 99.4%, p<0.001); this was mainly explained by the country where the study was conducted (p<0.001), the HL tool used (p = 0.002) and the country’s region (p<0.001).

**Fig 4:**
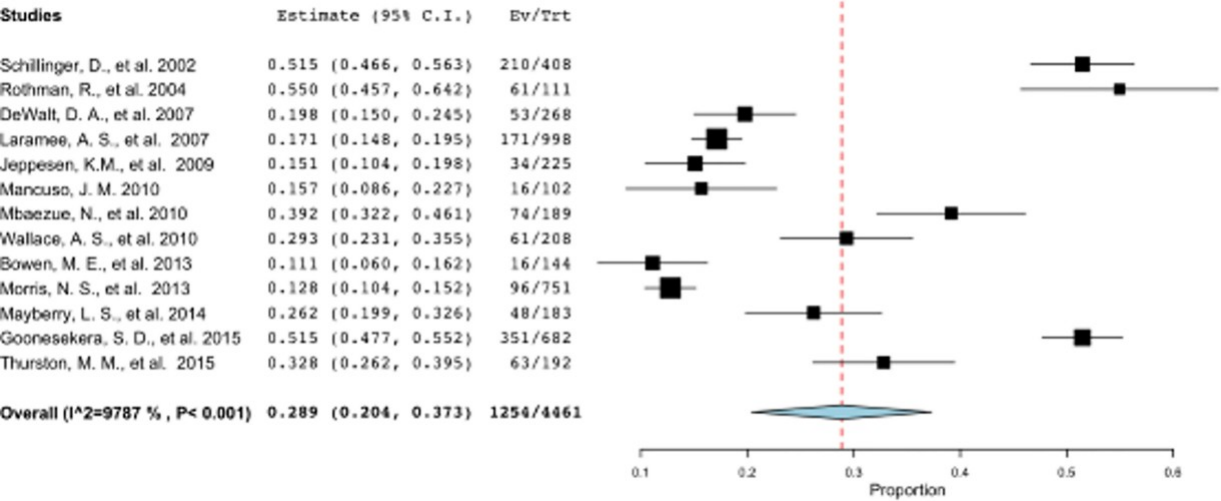
Meta-analysis of functional HL studies in the USA. (Refer Fig 4 Meta-analysis of functional HL studies in the USA.TIFF).

Most of the included studies (n = 18) were conducted in the USA. Thirteen of these studies measured functional HL specifically, these studies were included in a separate meta-analysis and presented in a forest plot in Fig 4. The pooled prevalence of functional limited HL in the USA was 28.9% (95% CI: 20.4–37.3) with heterogeneity score of 97.9%. Meta-regression analysis identified two factors that predicted this heterogeneity, the study setting (p = 0.005) and the proportion of participants with more the high school education (p = 0.009).

## Discussion

### Summary of findings

The global data on limited HL in patients with T2DM is limited. Final prevalence data presented in our systematic review came from only seven countries which were mainly middle to high-income countries. Whereas, nearly 79% of patients with T2DM live in low to middle income countries.[1] Many of these regions also struggle with low education levels thus further compounding the impact of low HL. From this systematic review, we could estimate that the prevalence of limited HL in these regions is at best 30%, but most probably higher.

In the USA, the pooled prevalence of patients with T2DM with limited HL is marginally lower than the proportion of the normal population with less than intermediate HL according to the 2003 NAAL (30% vs. 36%). [56] Similarly, in Canada, the proportion of adults with limited HL in the general population (60%) is higher compared to that of patients with T2DM (12.6 and 15.6%) [57,58] These findings correlated with our meta-regression analysis that patients surveyed in healthcare settings have a lower prevalence of limited HL compared with those surveyed in the community. We postulated that contact with healthcare systems and particularly healthcare professionals improve patients’ HL level.

There were seven different HL tools used by the studies included in this review. Older tools such as the TOFHLA and REALM tend to measure only one aspect of HL, the functional HL, while newer ones attempt to incorporate a multidimensional approach by assessing multiple aspects of HL such as print literacy, numeracy and in some cases oral literacy. [59] Multiple measurement tools would have inconsistent reporting of psychometric properties making a comparison of final results difficult. [60,61] This systematic review confirms the need for countries to measure the burden of limited HL in their patients with T2DM and to use one standardised tool. A standardised method of measuring HL would allow for a direct comparison of findings between countries. [62] Realising the importance of standardising method of HL measurement, HL researchers in Europe and Asia have taken the first step of validating and using one questionnaire translated and validated in the country’s local language.

The HLS-EU project used the HLS-EU-Q47 to measure HL in the general populations of eight countries across the European Union (EU) and Duong et al translated and culturally adapted the same instrument for use in six Asian countries. [63,64] The European study identified great differences in the proportion of limited HL in the general population of member countries, ranging from 28.7% in the Netherlands to more than double (62.1%) in Bulgaria. These results highlighted possible specific vulnerable groups within each country’s population. [64] A similar effort is needed on the assessment of HL in patients with T2DM. Since many diabetes care protocols and clinical practice guidelines are developed as an intercountry effort, collaborative effort in examining and comparing the burden of limited HL across the country would allow for countries to learn from each other.

### Limited functional HL in patients with T2DM in the USA

HL prevalence studies in patients with T2DM were done mostly in the USA. In our systematic review, 18 out of 29 were done in the USA and of these 13 studies measured functional HL. Healthcare providers in the USA need to be aware that almost one in three patients with T2DM they see would find it difficult to self-manage their condition and to make daily healthcare decisions. Patients with T2DM and limited HL have been shown to have less diabetes-specific knowledge [14,65] and to struggle with patient-provider communications and understanding of medical terminology. [66,67] Both knowledge and oral communication skills are important for empowering patients for self-management. These patients also have less desire to participate in shared decision-making. [34] When faced with such patients, healthcare providers should provide information in an easy-to-understand way and use the “teach-back” technique to reduce any chance of misunderstandings thus mitigating the impact of limited HL. [68]

Higher prevalence of limited HL was reported in patients surveyed in the community compared with those who attended primary care or hospitals. This finding highlighted the important role of HL in empowering patients to access and navigate healthcare systems. Furthermore, interactions with healthcare professionals may have led to the improvement of patients’ HL skills. These interactions exposed patients to common terminologies used in healthcare and healthcare professionals would have helped patients to understand and apply health information better. This finding supported current policy in the USA for the creation of more health-literate healthcare organisations, which would enable patients to access and benefit optimally from the health care services offered. [69]

The finding that the HL level is strongly associated with education level has been well described. Patients with higher education level benefit from the ability to understand their health needs, follow or read instructions, advocate for themselves and their families, and communicate effectively with health providers. [70] Furthermore, functional HL tools measured numeracy, reading and comprehension abilities, which are closely related to literacy skills. Interestingly, HL has been shown to be the mediating factor on the effect of education on health outcomes. [70,71] Unfortunately, healthcare providers will not be able to choose or improve patients’ educational attainment. Knowing our finding, healthcare providers could ensure this group of patients gets all the support they need to understand, appraise and apply health information in the process of managing their condition.

### Strengths and weakness

To the best of our knowledge, this review is the first to synthesise and summarise the burden of limited HL in patients with T2DM. Previous reviews concentrated on the instruments used to measure the HL levels and the economic burden of limited HL in the management of patients with T2DM. We searched through five electronic databases including an education database, ERIC. However, this review included only studies published in English, up to January 2017. The search may have excluded information in grey literature such as government reports and academic theses that were not published. We also found lack of data on the prevalence of inadequate HL in similar populations in these studies without T2DM

## Conclusions

The increasing burden of T2DM will exert greater pressure on healthcare systems across the world. Limited HL is a threat to patients’ empowerment and self-management. This review identified a high burden of limited HL in patients with T2DM with wide variations between countries. Currently, this observation is made based on published data from only a handful of countries. In the USA, one in three patients with T2DM has limited functional HL. Studies done in the community and in populations with less than high school education level reported higher limited HL prevalence. Further studies must explore the contextual factors before developing and implementing interventions to improve HL in these patients.

## Supporting information

S1 Table Search terms used in PubMed. (DOCX)

S2 Table PRISMA checklist. (DOC)
