# Correction: Prevalence of limited health literacy among patients with type 2 diabetes mellitus: A systematic review

**DOI:** 10.1371/journal.pone.0261430

**Published:** 2022-01-04

**Authors:** Adina Abdullah, Su May Liew, Hani Salim, Chirk Jenn Ng, Karuthan Chinna

There were errors in the extraction of numbers used to calculate the prevalence of limited health literacy, resulting in the incorrect extracted values for Souza, J. G., et al (2014), Kim, S. H. (2009), Chen, G. D., et al (2014), van der Heide, I., et al (2014), Aikens JE, Piette JD. (2009), Mancuso, J. M. (2010) and Wallace, A. S., et al (2010) in Table 1. Please see the corrected Table 1 here.

**Table 1:** Characteristics of included studies.

Country	Authors (year)	Year	Sample size	Main aims	Study design	Setting	Tool	Participants	Prevalence: % (n/N)
**Brazil**	De Castro, S. H., et al (2014)	NR	150	To assess the frequency of full and functional health illiteracy	Cross-sectional	Hospital outpatient	s-TOFHLA	Mean age = 58.5 years (SD 9.8), 52.4%, female, 28.4%—less than high school education.	26.7% (40/150)
Brazil	*Souza*, *J*. *G*., *et al (2014)*	*2012*	*129*	*To investigate the relationship between functional health literacy and glycaemic control in a sample of older patients*	*Cross-sectional*	*Hospital outpatient*	*SAHLPA-18*	*Mean age = 75*.*9 years (SD 6*.*2)*, *69*.*8%*, *female*, *82*.*9%—having less than a high-school diploma*.	*56*.*6%* *(73/129)*
**Canada**	Al Sayah, F., et al (2015)	NR	154	To examine the relationship of inadequate health literacy (HL) with changes in depressive symptoms, health-related quality of life and cardiometabolic outcomes in patients recently screened positive for depression.	Longitudinal	Primary care clinics	BHLS	Mean age = 58.1 years (SD 9.4), 55.8%, female, 13.7%—less than high school	15.6% (24/154)
**Canada**	Sayah, F. A., et al (2016)	2013	1948	To examine the association of health literacy (HL) with changes in health-related quality of life (HRQL)	Longitudinal	Primary care clinics	BHLS	Mean age = 65.6 years (SD 11.4), 45%, female, 14.2%—less than high school education.	12.5% (244/ 1948)
**Marshall Island**	Bohanny, W. M., et al (2013)	2009	150	To explore the relationships among health literacy, self-efficacy, and self-care behaviors	Cross-sectional study	Primary care clinics	s-TOFHLA	Mean age = 52.7 years (SD 10.5), 53.3%, female, 44%—less than high school	24% (36/150)
**South Korea**	*Kim*, *S*. *H*. *(2009)*	*2007*	*53*	*To investigate the relationships of health literacy to chronic medical conditions and the functional health status*	*Cross-sectional study*	*Community based*	*Korean Functional Health Literacy test*	*Mean age = 67*.*2 years*, *63*.*3%*, *female with limited literacy*.	*37*.*7%* *(20/53)*
**Switzerland**	Franzen, J., et al (2014)	2011	493	To measure functional HL among persons having type 2 diabetes and to investigate the relationship between functional HL and health care costs and utilization	Cross-sectional study	Insurer’s database	BHLS	Mean age = 67.5 years, 51.5% belongs to 65-70-year-old group, n = 391, 32.7%, female	7.3% (36/493)
**Switzerland**	Mantwill, S., et al (2015)	2012	391	To determine the relationship between health literacy and three years of medication costs	Cross-sectional study	Insurer’s database	BHLS	Mean age = 63.8 years (SD 6.1), 32.2%, female, 13.1%—less than high school education.	8.7% (34/391)
**Taiwan**	*Chen*, *G*. *D*., *et al (2014)*	*2012*	*467*	*To demonstrate the interaction of health literacy and understanding of health education and instructions in achieving glycemic control*	*Cross-sectional study*	*Hospital outpatient*	*MHLS*	*Mean age = 68*.*3 years (SD 7*.*4)*, *70*.*2%*, *female with limited literacy*, *61*.*5%—less than compulsory education*	*47*.*3% (221/467)*
**Taiwan**	Tseng, H.-M., et al (2017)	NR	232	To explore the mechanisms through which HL is associated with the health outcome of diabetic care	Cross-sectional study	Hospital outpatient	NVS	Mean age = 58.1 years (SD 9.49), 44.8%, female, 90.1%—secondary education and less	76.3% (177/232)
**Netherlands**	*van der Heide*, *I*., *et al (2014)*	*2010*	*1676*	*To investigate whether diabetes knowledge can account for part of the relation between health literacy and diabetes self-management behaviour*	*Cross-sectional study*	*Primary care clinics*	*BHLS*	*65–74 years group (31*.*7%)*, *49*.*6%*, *female*, *44*.*9% low level of education*	*9*.*8% (164/1676)*
**United States of America (USA)**	Schillinger, D., et al (2002)	2000	408	To examine the association between health literacy and diabetes outcomes	Cross-sectional study	Primary care clinics	s-TOFHLA	Mean age = 62.7 years (SD 10.9), 58%, female, 46%—some high school education or less	51.5% (210/408)
**United States of America (USA)**	Rothman, R., et al (2004)	2000	111	To examine the role of literacy in patients with poorly controlled diabetes who were participating in a diabetes management program that included low-literacy-oriented intervention	Cross-sectional study	Hospital internal medicine clinic	REALM	Mean age = 60 years, 56%, female has limited health literacy, 82%—less than high school education	55% (61/111)
**United States of America (USA)**	Laramee AS, et al (2007)	2005	998	To determine the prevalence of limited literacy in diabetic patients with heart failure (HF) compared to those with diabetes and no HF	Cross-sectional study	Primary care clinics	s-TOFHLA	Mean age = 65 years (22–93), 54%, female, 25%—less than high school graduate.	17.1% (171/998)
**United States of America (USA)**	DeWalt, D. A., et al (2007)	2005	268	To examine the relationship between literacy and trust, self-efficacy, and participation in medical decision making	Cross-sectional study	Hospital outpatient	REALM	Mean age = 62 years (SD 10), 57%, female with limited health literacy .	19.8% (53/268)
**United States of America (USA)**	*Aikens JE*, *Piette JD*. *(2009)*	*2007*	*1376*	*To determine how patients’ beliefs about antihyperglycemic and antihypertensive medications relate to medication underuse and health status*.	*Cross-sectional study*	*Primary care clinics*	*BHLS*	*Mean age = 55*.*3 years (SD 11*.*8)*, *61*.*6%*, *female*, *21*.*6%—less than high school*	*38*.*2% (525/1376)*
**United States of America (USA)**	Jeppesen KM, et al (2009)	2007	225	To identify questions that could best indicate to a clinician that a patient may have low or marginal health literacy	Cross-sectional study	Primary care clinics	s-TOFHLA	Mean age = 53.8 years (SD 12.8), 68.4%, female, 44.9%—less than high school education.	15.1% (34/225)
**United States of America (USA)**	*Mancuso*, *J*. *M*. *(2010)*	*NR*	*98*	*To examine if health literacy and patient trust in one’s health-care provider impact glycemic control in an uninsured population*	*Cross-sectional study*	*Primary care clinics*	*TOFHLA*	*Mean age = 52 years (SD 9*.*1)*, *60*.*8%*, *female*, *33*.*3%—Less than high school education*.	*37*.*8%* *(37/98)*
**United States of America (USA)**	Mbaezue N, et al (2010)	2005	189	To examine the relationship between health literacy and self-monitoring of blood glucose (SMBG)	Cross-sectional study	Hospital-based clinic	s-TOFHLA	Mean age = 51.2 years (SD 10.0), 58.7%, female, 32.3%—less than high school education.	39.2% (74/189)
**United States of America (USA)**	*Wallace*, *A*. *S*., *et al (2010)*	*2008*	*195*	*To examine whether demographic characteristics*, *insurance status*, *literacy*, *duration of diabetes*, *and intensity of care management were associated with PACIC ratings*	*Cross-sectional study*	*Hospital diabetes clinic*	*s-TOFHLA*	*Mean age = 58 years (range*: *23–85)*, *64%*, *female*, *34%—Less than high school education*.	*31*.*3% (61/195)*
**United States of America (USA)**	Bauer, A. M., et al (2013)	2006	1366	To determine whether health literacy limitations are associated with poorer antidepressant medication adherence.	Cohort study	Insurer’s database	BHLS	Mean age = 58.7 years (SD 10.5), 59.9%, female with limited Health literacy, 28.1%—less than high school	72% (984/1366)
**United States of America (USA)**	Bowen, M. E., et al (2013)	2009	144	To describe the association among numeracy, total energy, and macronutrient intake	Cross-sectional study	Primary care clinics	REALM	Median age = 56 years, 53%, female, 26%—high school education or less	11.1% (16/144)
**United States of America (USA)**	Morris, N. S., et al (2013)	2007	751	To evaluate the stability of health literacy over time	Longitudinal study	Primary care clinics	s-TOFHLA	12% belong to 70 years old age group, 53%, female with limited health literacy, 70%—Some high school education.	12.8% (96/751)
**United States of America (USA)**	Mayberry, L. S., et al (2014)	2012	183	To assess whether obstructive family behaviors had a stronger relationship with worse glycemic control among patients with limited HL than among those with adequate health literacy	Cross-sectional study	Hospital outpatient	s-TOFHLA	Mean age = 51.2 years (SD 10.6), 70%, female, 64%—less than high school education	26.2% (48/183)
**United States of America (USA)**	Thurston, M. M., et al (2015)	2013	192	To determine (1) if a relationship exists between health literacy and self-reported or objectively measured medication adherence and (2) which aspect or aspects of medication nonadherence are most associated with health literacy	Cross-sectional study	Primary care clinics	s-TOFHLA	Mean age = 54.4 years (SD 10.3), 56.8%, female, 64.6%—less than high school education	32.8% (63/192)
**United States of America (USA)**	Sayah, F. A., et al (2015)	2010	343	To examine the associations between inadequate health literacy and behavioral and cardiometabolic parameters	Cross-sectional study	Primary care clinics	BHLS	Mean age = 57.4 years (SD 10.11), 68%, female, 25%—less than high school education	23.9% (82/343)
**United States of America (USA)**	Goonesekera, S. D., et al (2015)	2012	682	To examine racial/ethnic differences in receipt of hypoglycaemic medications and glycaemic control	Cross-sectional study	Community based	s-TOFHLA	56% belongs to less than 65 years old group, 51%, female, 18%—less than high school.	51.5% (351/682)
**United States of America (USA)**	Fan, J. H., et al (2016)	2014	208	To investigate the relationship between health literacy and overall medication nonadherence, unintentional nonadherence, and intentional nonadherence	Cross-sectional study	Primary care clinics	BHLS	Mean age = 53 years (SD10.9), 70.9%, female, 19%—had less than a high school education	63.5% (132/208)
**United States of America (USA)**	Nelson, L. A., et al (2016)	NR	80	To examine the relationship between patient factors and engagement in an mHealth medication adherence promotion intervention for low-income adults	Intervention study	Hospital outpatient	BHLS	Mean age = 50.1 years (SD 10.5), 54%, female, 56.3%—less than a high school degree	46.3% (37/80)

The Results section has also been affected by the errors in the extracted values. In the Included Studies subsection of the Results, there are errors in the second paragraph. The corrected paragraph should read: The study with the highest reported prevalence of limited health literacy (76.3%) was conducted to determine the mechanism through with health literacy exerted its influence on health outcomes related to diabetes care. It was a cross-sectional study involving 232 patients with T2DM attending regional hospital in Northern Taiwan. Health literacy was assessed using NVS. The mean age of the participants was 58.02 years (SD 9.49), 44.8% of participants were female, and 38.4% had received primary education or below. [55]

The Pooled prevalence of limited HL: A meta-analysis subsection of the Results has also been affected and have been updated as a result. The corrected section should read: The pooled global prevalence of limited health literacy was 32.5% (95% CI: 24.9–40.1). Meta-analysis of all included studies yielded high heterogeneity (I2 = 99.3%, p < 0.001); which could primarily be explained by the country in which the study was conducted (p<0.001), the health literacy tool used (p = 0.002), participants’ education levels (p<0.001), and the setting where the study was conducted (p<0.001). Most of the included studies (n = 18) were conducted in the USA. Thirteen of these studies measured functional HL specifically, these studies were included in a separate meta-analysis and presented in a forest plot in Fig 4. The pooled prevalence of functional, limited health literacy in the USA was 34.5% (95% CI: 24.1–45), with a heterogeneity score of 99.1%. Meta-regression analysis identified two factors that predicted this heterogeneity, the study setting (p = 0.005) and the proportion of participants with more the high school education (p = 0.009).

Figs 2 and 4 were also affected and have been updated as a result, please see the corrected figures here.

**Fig 2:**
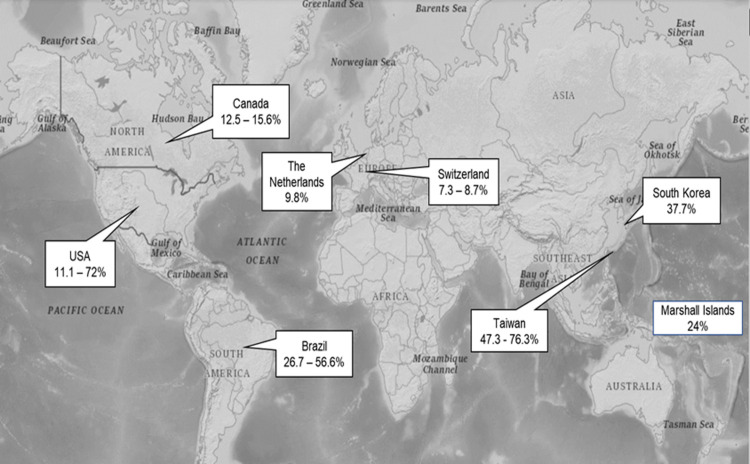
Worldwide prevalence of limited HL in patients with type 2 DM. (Refer Fig 2_Worldwide prevalence of limited HL.TIFF).

**Fig 4:**
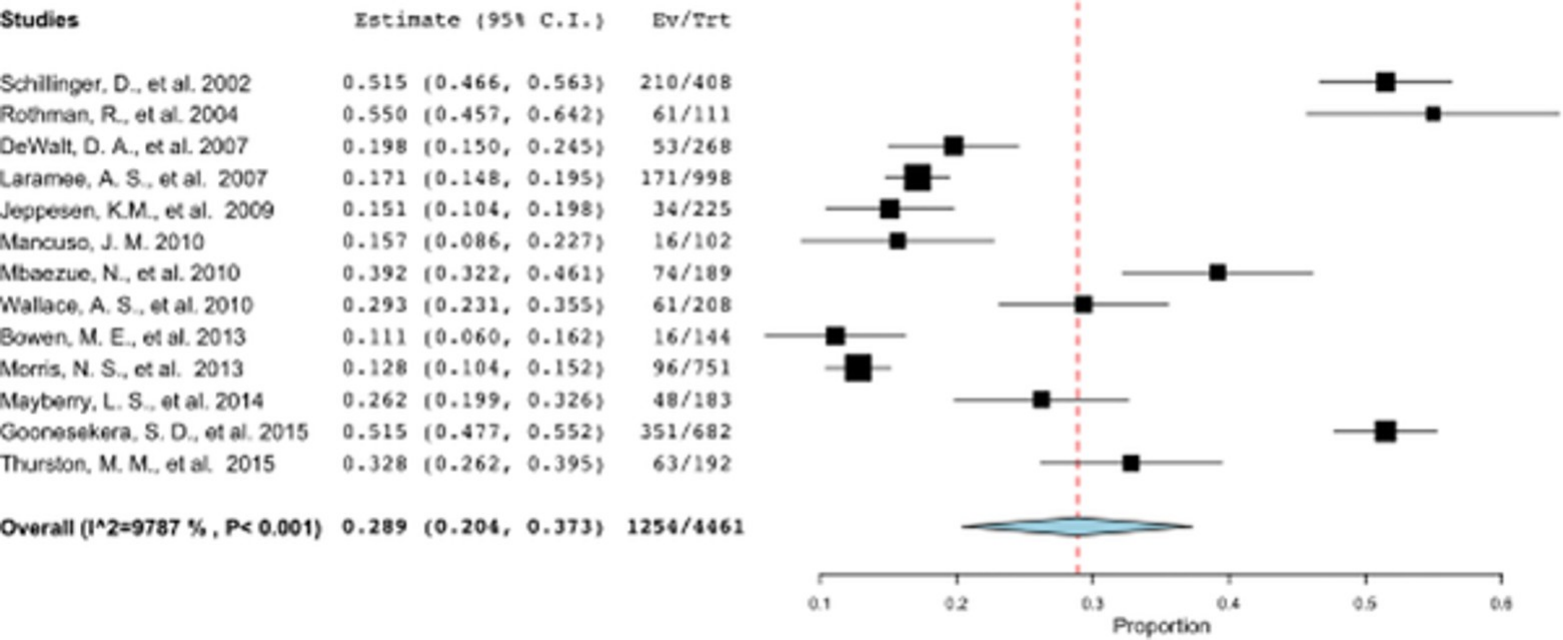
Meta-analysis of functional HL studies in the USA. (Refer Fig 4 Meta-analysis of functional HL studies in the USA.TIFF).
