# Supplementary material for: Prevalence of limited health literacy among patients with type 2 diabetes mellitus: A systematic review
Source: PLoS One. 2019 May 7;14(5):e0216402. doi: 10.1371/journal.pone.0216402 (PMC6504081; doi:10.1371/journal.pone.0216402)
Supplement: S1 Table — (DOCX) [file pone.0216402.s001.docx]

S1 Table: Search terms used in PubMed

| Concepts | Search terms in PubMed |
| --- | --- |
| Health Literacy | ((((((((((((((((((((health[Title]) AND literacy[Title])) OR ((“health literacy” OR “health literate” OR “medical literacy”))) OR (((functional[Text Word]) AND health[Text Word]) AND literacy[Text Word])) OR numeracy) OR ((((low literate[ti] OR low literacy[ti] OR literacy[ti] OR illiteracy[ti] OR literate[ti] OR illiterate[ti] OR reading[mh] OR comprehension[mh]) AND (health promotion[major] OR health education[major] OR patient education[major] OR Communication Barriers[major] OR communication[major:noexp] OR Health Knowledge, Attitudes, Practice[major] OR attitude to health[major]))))) OR (((comprehension[major] AND educational status[major])))) OR (((family[ti] AND literacy[ti])))) OR ((((“drug labeling” OR Prescriptions[mh]) AND (“comprehension” OR “numeracy”))))) OR ((((cancer[ti] OR diabetes[ti]) AND (literacy[ti] OR comprehension[ti]))))) OR “adult literacy”) OR “limited literacy”) OR “patient understanding”[Title]) OR (((self care[major] AND perception[mh])))) OR (((comprehension AND food labeling[mh])))) OR (((comprehension AND informed consent)))) OR (((comprehension AND insurance, health))))) OR health literacy[MeSH Terms]) OR Patient Medication Knowledge[MeSH Terms] |
| Type 2 Diabetes Mellitus | (((((((((((((((non insulin* AND depend*) OR noninsulin* AND depend*)) OR NIDDM[Text Word]) OR dm2[Text Word]) OR (((((insulin* AND resistanc*[Text Word]) OR impaired glucose toleranc*[Text Word]) OR glucose intoleranc*[Text Word]) OR "Insulin Resistance"[Mesh]) OR "Diabetes Mellitus, Type 2"[Mesh])) OR ((((diabetes mellitus[Title/Abstract]) OR diabete*[Text Word])) AND ((obesity[Text Word]) OR obes*[Text Word])))) OR (obes* AND adj diabet*[Text Word])) OR dm2[Text Word]) OR NIDDM[Text Word]) OR ((non insulin* AND depend*) OR noninsulin* AND depend*)) OR (((((typ* AND 2) OR typ*II OR (typ* AND ii)) AND adj diabet*)))) OR (('keto*resist*'[Text Word] OR non *keto*') AND adj diabet*[Text Word]))) NOT ((diabet* AND insipidus[Text Word]) OR diabetes insipidus) |
